# Supplementary material for: Designer exosomes produced by implanted cells intracerebrally deliver therapeutic cargo for Parkinson’s disease treatment
Source: Nat Commun. 2018 Apr 3;9:1305. doi: 10.1038/s41467-018-03733-8 (PMC5880805; doi:10.1038/s41467-018-03733-8)
Supplement: Supplementary file 1 — Supplementary Information(PDF 538 kb) [file 41467_2018_3733_MOESM1_ESM.pdf]

Supplementary information

Designer exosomes produced by implanted cells intracerebrally deliver therapeutic cargo for Parkinson's disease treatment

Ryosuke Kojima, Daniel Bojar, Giorgio Rizzi, Ghislaine Charpin-El Hamri, Marie Daoud-El Baba, Pratik Saxena, Simon Auslaender, Kelly R. Tan, Martin Fussenegger

Inventory of this supplementary information

Supplementary Methods ······ 2

Reporter gene assays ······ 2

ELISA assay ······ 2

In vivo experiments ······ 3

Supplementary figures and tables

Supplementary Figure 1. Result of gene screening of potential exosome production boosters ······ 5

Supplementary Figure 2. Additional data to support the boosting of exosome production ······ 6

Supplementary Figure 3. Detailed result of nanoparticle tracking analysis (NTA) ······ 7

Supplementary Figure 4. Assay of the exosome production booster in several different cell lines ······ 8

Supplementary Figure 5. Assay to show mRNA transfer is mediated by vesicle components ······ 9

Supplementary Figure 6. Comparison of the effect of Cx43 WT and Cx43 S368A ······ 9

Supplementary Figure 7. Protection against neurotoxicity caused by LPS administration by therapeutic catalase mRNA delivery ······ 10

Supplementary Figure 8. CD63-nluc assay in mouse blood ······ 10

Supplementary Figure 9. Nluc assay in vivo with a macro encapsulator ······ 11

Supplementary Figure 10. Biodistribution of mRNA delivery ······ 12

Supplementary Table 1. Description of the plasmids used in this study ······ 13

Supplementary Table 2. Table of the oligonucleotides used for cloning ······ 14

Supplementary references ······ 15

Supplementary Methods

Reporter gene assays

Exosome production assay with the reporter CD63-nluc

The supernatant of exosome producer cells was collected 24-36 hours after medium change following the transfection. After stepwise centrifugation to remove cells and cell debris from the sample (300xg 5 min, 2000xg 10 min, 10000xg for 30 min), the supernatant was assayed. The protocol for each figure is as follows.

Supplementary Fig. 1: 200 ng of pDB30 (P_{hCMV}-CD63-nluc-pA) and 200 ng of potential exosome production boosters (pEYFP-C1 was used as a control) per well were co-transfected into HEK-293T cells in a 24-well plate (the amount of DNA was made up to 500 ng with pcDNA3.1+). At 16 hr after transfection, the medium was changed to fresh DMEM. At 24 hrs after the medium change, nluc in the supernatant was measured.

Supplementary Fig. 4. HeLa cells, hMSCs-TERT and CHO-K1 cells were transfected with 40 ng of pDB30 (P_{hCMV}-CD63-nluc-pA) together with 120 ng of pDB60 (P_{hCMV}-STEAP3-IRES-SDC4-IRES-nadB fragment-pA) (for +enhancer condition) or the same amount of pEYFP-C1 (for –enhancer condition) per well in a 96-well plate. At 16 hrs after transfection, the medium was changed to fresh DMEM. At 24 hrs after the medium change, nluc in the supernatant was measured.

mRNA transfer assay

Supernatant of exosome producer cells was collected 24-48 hours after medium change after transfection. This supernatant was transferred to target cells prepared in 48- or 24-well plates (60-80 % confluency) after centrifugation to remove cells and cell debris. After 24 hours, the receiver cells were trypsinized, spun down, and suspended in PBS (25 µL/cells from each well of a 48-well plate). 7.5 µL of the suspension were pipetted into a 384-black well plate, and 7.5 µL quantification reagent (0.15 µL Nano-Glo® Luciferase Assay Substrate per 7.5 µL of Nano-Glo® Luciferase Assay Buffer) were added. After 5 min, luminescence measurements were performed at 25°C on a Tecan Infinite® M200 PRO with an integration time of one second per well. The luminescence value was quantified as relative light unit (RLU). The specific procedure for each figure was as follows.

Supplementary Fig. 5: See corresponding figure legend.

Supplementary Fig. 6: The same protocol as for Fig. 2c was used except for the difference in the cytosolic delivery helper. pDB68 (P_{hCMV}-Cx43 S368A-pA) was used for "Cx43 S368A" condition, pDB67 (P_{hCMV}-Cx43-pA) was used for "Cx43 WT" condition, and pEYFP-C1 was used for "no Cx43" condition.

ELISA assay

Protein levels in the eluted exosomes obtained as described under 'Nanoparticle Tracking Analysis' were measured by means of ELISA. Kits from Chemie Brunschwig were used for the measurement of CD9 (Art. No. CSB-EL004969HU), TSG101 (Art. No. CSB-EL025125HU) and HSP90B1 (Art. No. CSB-E08551h), according to the manufacturer's instructions. Protein levels in the samples were interpolated from standard curves.

Rescue of LPS-induced neurotoxicity *in vitro*

Cell culture supernatant containing catalase-mRNA-bearing exosomes was prepared using the same method as described for

the assay using 6-OHDA. The conditioned medium was added to a co-culture of Neuro2A cells and murine microglial BV2 cells seeded in a 96-well plate (200 µL/well) in triplicate (1.88 x 10^4 cells of Neuro2A cells and 3.76 x 10^3 cells of BV2 cells (ratio of 5:1) were seeded per well of a 96-well plate one day before this medium transfer). After 24 hrs, 0-20 µg/mL of LPS (from E. coli O111:B4, InvivoGen) in DMEM were added to the cells (final: 0-1 µg/mL). After 24 hrs, the medium was replaced with fresh DMEM containing 10% CCK-8 assay solution, and CCK-8 assay was performed according to the manufacturer's protocol. The measurement of absorbance was performed at 450 nm with an EnVision 2104 plate reader. In setting up this experiment, we were guided by a previous report[1].

In vivo experiment

Assay of penetration of exosomes into blood vessels

Supplementary Fig. 8: HEK-293T cells in a 15 cm dish were transfected with 6600 ng of pDB30 (P_{hCMV}-CD63-nluc-pA), 12500 ng of pDB60 (P_{hCMV}-STEAP3-IRES-SDC4-IRES-nadB fragment-pA), and 1100 ng of pRVG-Lamp2b (P_{hCMV}-RVG-Lamp2b-pA) (2 dishes were prepared, approx. 3.6x10^7 cells in total; plasmid amount is per 15 cm dish). After overnight transfection, the cells were trypsinized (2 dishes/group were combined), spun down, and re-suspended in 1.8 mL DMEM without FBS. The cell suspension was put on ice and mixed with 1.8 mL of ice-cold Matrigel (Corning). 400 µL of the cell/Matrigel mixture was injected s.c. into C57BL/6J mice (8 weeks of age, female, total 8 mice). 24 hrs later, blood was collected and serum was prepared. Luminescence was measured with a Tecan Infinite M200 PRO (7.5 µL serum + 7.5 µL assay solution per well of a 384-well plate).

Supplementary Fig. 9a: The same exosome producer cells as used in Supplementary Fig. 7 (1x10^6 cells/1 implant) were encapsulated in a macro encapsulator. The encapsulator containing the exosome producer cells was embedded s.c. on the back of C57BL/6 mice. After 24 hrs, blood was collected and serum was prepared. Luminescence emission from the serum was measured with a Tecan Infinite M200 PRO (7.5 µL serum + 7.5 µL assay solution per well in a 384-well plate).

Nanoluc mRNA delivery

Supplementary Fig. 10: HEK-293T cells in a 15-cm dish were transfected with the following plasmids (2 dishes were prepared per condition; plasmid amount is per 15 cm dish). With device: 2750 ng of pSA465 (P_{hCMV}-CD63-L7Ae-pA), 5500 ng of pDB60 (P_{hCMV}-STEAP3-IRES-SDC4-IRES-nadB fragment-pA), 1100 ng of pRVG-Lamp2b (P_{hCMV}-RVG-Lamp2b-pA), 9750 ng of pSA462 (P_{hCMV}-nluc-C/D_{box}-pA), and 1100 ng of pDB68 (P_{hCMV}-Cx43 S368A-pA). Without device: 10450 ng of pEYFP-C1 (P_{hCMV}-EYFP-pA), 9750 ng of pSA462 (P_{hCMV}-nluc-C/D_{box}-pA). After overnight transfection, the cells were trypsinized (2 dishes/group were combined), spun down, and re-suspended in 1.8 mL DMEM without FBS. The cell suspension was put on ice and mixed with 1.8 mL of ice-cold Matrigel (Corning). 400 µL of the cell/Matrigel mixture was injected s.c. into a C57BL/6J mice (8 weeks of age, female, total 8 mice). 48 hrs later, the mice were killed by overdose anesthesia and whole-body perfusion was performed with PBS. The brain, spleen, liver of each mouse were removed and put into 500 µL (for brain and liver) or 300 µL (for spleen) of DPBS containing 1% protease inhibitor cocktail (Sigma). The tissue was mechanically homogenized and centrifuged at 9000xg for 20 min at 4°C. The supernatant was used for nluc assays (7.5 µL of the supernatant + 7.5 µL of assay solution) on a Tecan Infinite M200 Pro plate reader. The protein concentration of the supernatant was measured by Bradford assay (Quick Start Bradford 1x Dye Reagent, Bio-Rad), and the luminescence value was normalized by the amount of protein (RLU/mg protein)

Supplementary Fig. 9b: For the "with device, macro implant" condition, HEK-293T cells in a 10-cm dish were transfected

with 1500 ng of pSA465 (P_{hCMV}-CD63-L7Ae-pA), 3000 ng of pDB60 (P_{hCMV}-STEAP3-IRES-SDC4-IRES-nadB fragment-pA), 600 ng of pRVG-Lamp2b (P_{hCMV}-RVG-lamp2b-pA), 5300 ng of pSA462 (P_{hCMV}-nluc-C/Dbox-pA), and 600 ng of pDB68 (P_{hCMV}-Cx43 S368A-pA) After 8 hrs of transfection, $1x10^6$ cells (per implant) were encapsulated in the macro encapsulator. The macroencapsulated system containing the exosome producer cells was embedded s.c. on the back of C57BL/6 mice. After 48 hrs, the mice were killed with an overdose of anesthesia, and whole-body perfusion was conducted with an excess amount of PBS. Luminescence of the brain homogenate was measured with the same method as for Fig. 3d. Luminescence values were normalized by protein concentration as above, and also by cell number of the implanted exosome-producer cells (RLU/mg protein/sender cells. Other conditions ("without device, Matrigel condition" and "with device, Matrigel condition") were the same as for Fig. 3d.

Supplementary Figures

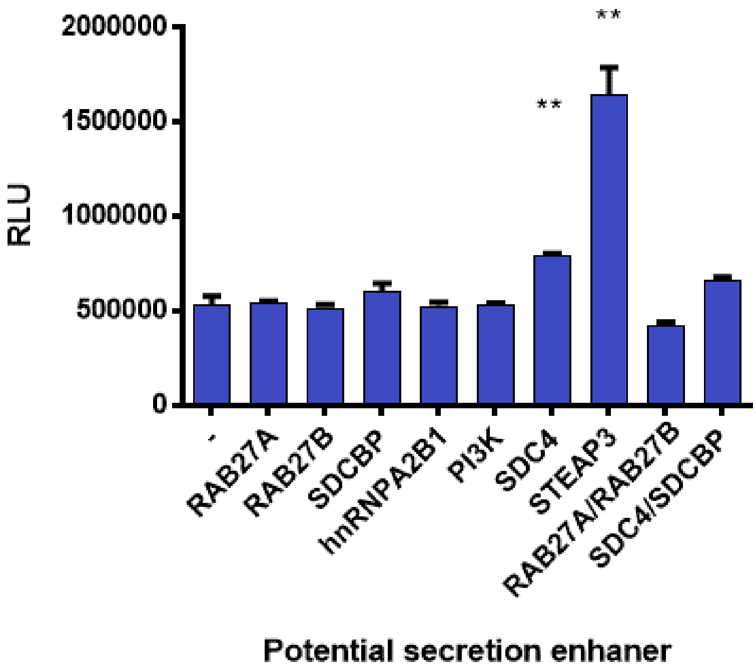

Supplementary Figure 1. Result of gene screening of potential exosome production boosters. CD63-nluc (pDB30 P_{hCMV}-CD63-nluc-pA) was co-transfected with potential exosome production boosters in HEK-293T cells, and nluc activity in the supernatant was assayed after stepwise centrifugation to remove live cells, dead cells and cell debris. For RAB27A: pDB11 (P_{hCMV}-RAB27A-pA), for RAB27B: pDB17 (P_{hCMV}-RAB27B-pA), SDCBP: pDB14 (P_{hCMV}-SDCBP-pA), hnRNPA2B1: pDB18 (P_{hCMV}-hnRNPA2B1-pA), PI3K: pRK50 (P_{hCMV}-PI3K-pA), SDC4: pDB13 (P_{hCMV}-SDC4-pA), STEAP3: pDB12 (P_{hCMV}-STEAP3-pA), RAB27A/27B: co-transfection of pDB11 and pDB17. SDC4/SDCBP: co-transfection of pDB13 and pDB14. Data shown represent mean ± SEM of three independent experiments (n = 3). **$p<0.01$, two-tailed Student's t-test (others: $p>0.05$) (compared to – condition). Note that the ratio of the plasmids encoding CD63-nluc and the potential exosome production booster is different from Figure 1b. This should be the reason for the differences in fold increase of nluc activity. Based on these results, STEAP3 and SDC4 were chosen as the components of exosome production booster. The NadB fragment was unexpectedly cloned into the 3rd cistron of pDB60 (P_{hCMV}-STEAP3-IRES-SDC4-NadB fragment-pA, and we found that it increased the signal from CD63-nluc. Later we confirmed its activity by cloning the NadB fragment into pcDNA3.1(+) (pRK 246, P_{hCMV}-NadB fragment-pA) (Fig. 1b). See plasmid table for detailed information.

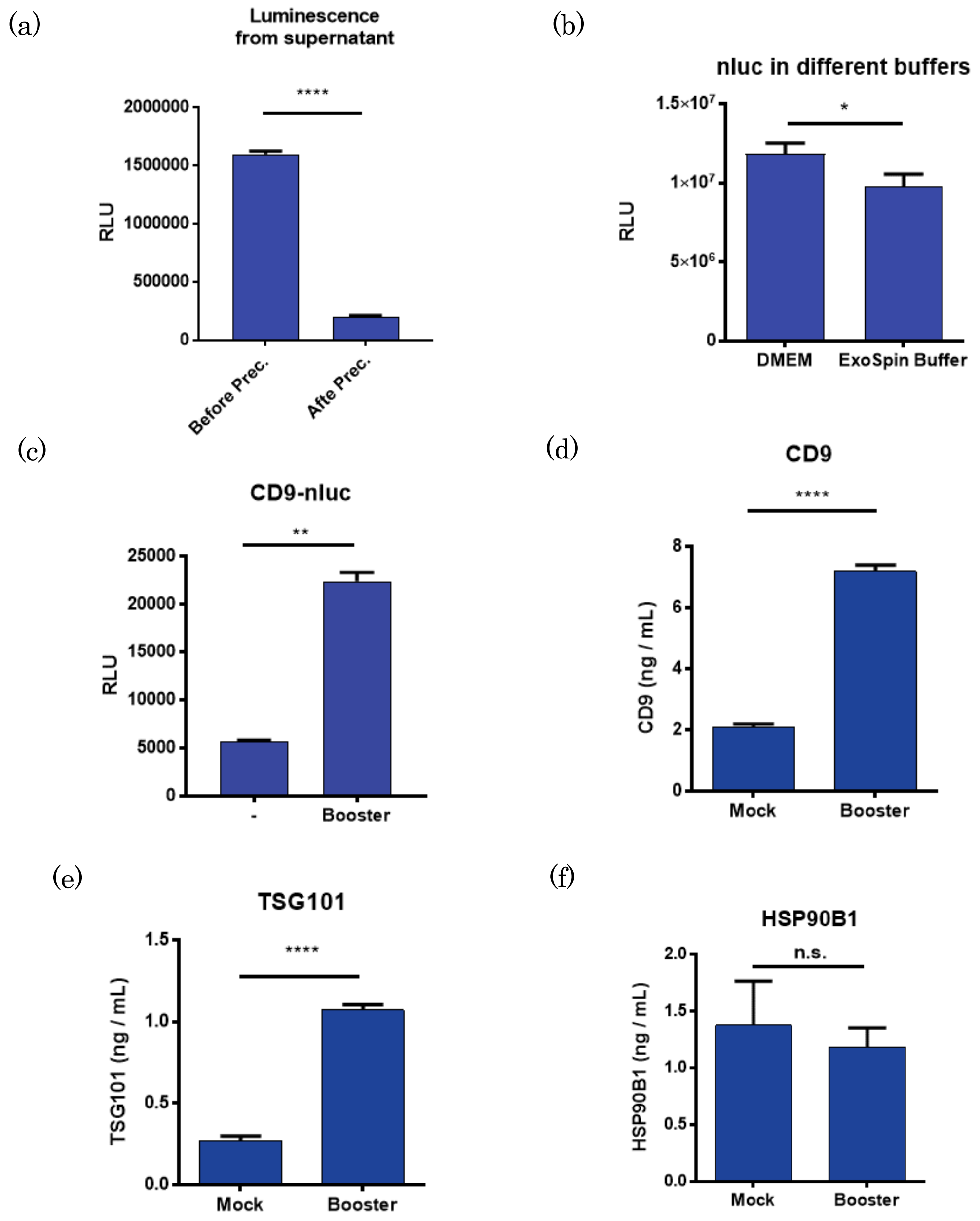

Supplementary Figure 2. Additional data to support the enhancement of exosome production (a) An assay to show that CD63-nluc luminescence signal from the cell culture supernatant originates mostly from vesicle components. First, exosome producer HEK-293T cells were transfected with pDB30 (P_{hCMV}-CD63-nluc-pA) and pDB60. The cell culture supernatant was collected and centrifuged at 300xg and 20000xg in a stepwise manner to remove live cells, dead cells, and cell debris (The collected supernatant was called "Before prec."). This supernatant was further treated with the Exo-spin kit (Cell Guidance Systems) to precipitate and purify the exosomes (this treated supernatant was called "after prec"). Nluc activity of "Before prec." and "After prec" was compared. The results suggest that most of the CD63-nluc signal is indeed from vesicle components. Note: we compared nluc activity in DMEM and ExoSpin buffer, and confirmed that the buffer components affect nluc activity only slightly in (b). (c) Luminescence assay using a CD9-nluc fusion. Exosome producer HEK-293T cells were transfected with pDB96 (P_{hCMV}-CD9-nluc-pA) and pDB60 (for the control, pEYFP-C1 was used). The

luminescence signal in the cell culture supernatant was assayed with the same protocol as in the assay with CD63-nluc (Fig.1b). (d) Result of CD9 (exosomal transmembrane protein) ELISA assay. The cell culture supernatant of exosome producer cells with or without the exosome production booster was assayed. (e) Result of TSG101 (soluble exosomal protein) ELISA assay. (f) Result of HSP90B1 ELISA assay (cytosolic marker as a control). We confirmed increases of CD9 and TSG101 in the vesicle fraction, but there was no increase of cytosolic HSP90B1.

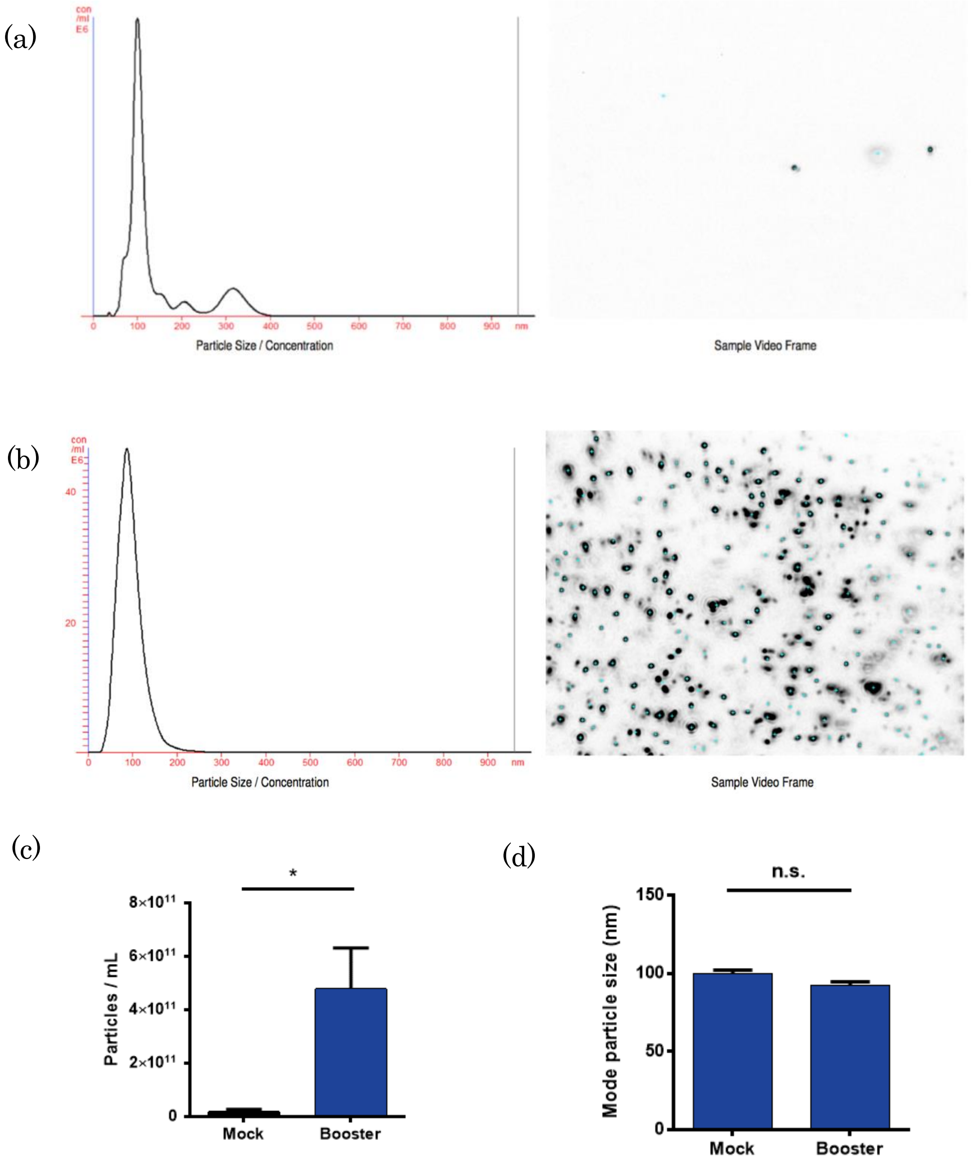

Supplementary Figure 3. Detailed data of the Nanoparticle Tracking Analysis (NTA). HEK-293T cells were transfected with pDB30 (P_{hCMV}-CD63-nluc-pA) and pDB60 (P_{hCMV}-STEAP3-IRES-SDC4-NadB fragment-pA) (for control, pEYFP-C1 instead of pDB60), and the engineered exosomes secreted from the cells were purified as described in the methods section. Biological triplicates were prepared for each condition. Representative data is shown for (a) and (b), and the averages of triplicate measurements are shown in (c) and (d). (a,b) Raw data of NTA assay. ((a) Result with control sample (mock) (b) Result with production booster.) Left: size distribution

and concentration of the purified exosomes (The merged graph of (a) and (b) is shown in Fig. 1c), Right: Sample video frame taken by Nanosight LM10 equipped with a CCD camera (c) Average concentration of the particles was compared between the control samples and the samples with exosome production booster. (d) Average size of particles detected in the NTA assay. Error bars represent ± SEM (n=3). *p<0.05, n.s.: p>0.05. two-tailed Student's t-test.

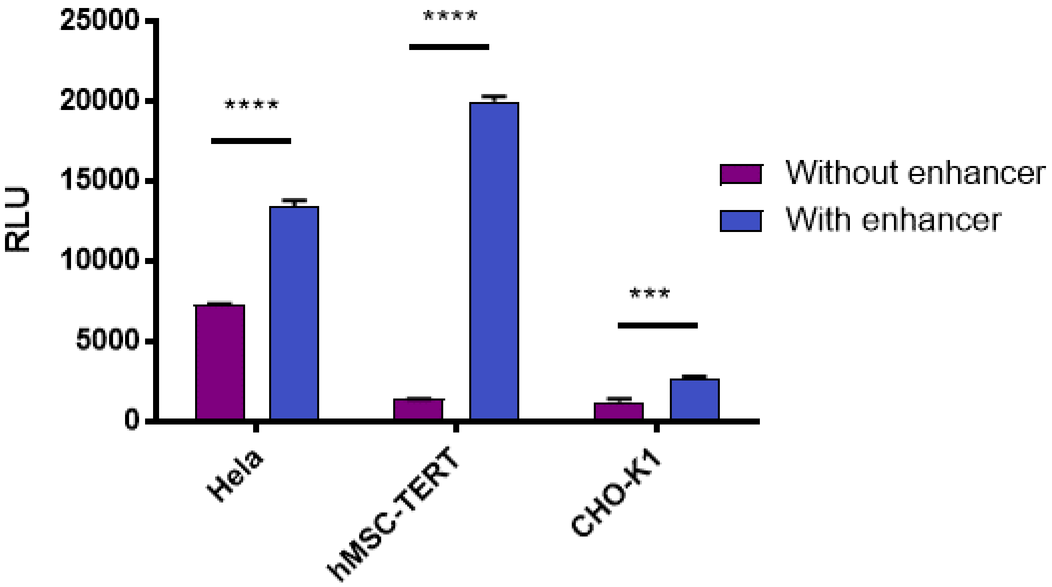

Supplementary Figure 4. Assay of the exosome production booster (pDB60) in several different cell lines. Each cell line was transfected with pDB30 (P_{hCMV}-CD63-nluc-pA) together with pDB60 (+enhancer condition) or pEYFP-C1 (-enhancer condition). Nluc activity in the supernatant was assayed after stepwise centrifugation to remove live cells, dead cells and cell debris. Error bars represent ± SEM of three independent experiments (n=3). ***p<0.001, ****p<0.0001, two-tailed Student's t-test.

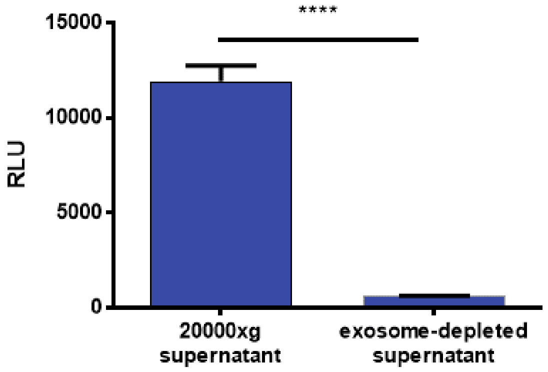

Supplementary Figure 5. An assay to show that mRNA transfer is indeed mediated by vesicle components. The exosome-producer HEK-293T cells bearing nluc mRNA were prepared with the same method as in Fig. 2b (full device), and the supernatant was centrifuged at 300xg and 20000xg to remove live cells, dead cells, and cell debris. This supernatant was used as "20000xg supernatant". This supernatant was further treated with Buffer A from the Exo-spin kit to precipitate all exosomes. The resulting supernatant was used as the "exosome-depleted supernatant". Both supernatants were applied to recipient HEK-293T cells expressing CHRNA7, and the nluc activity of the recipient cells was assayed (note that there was no apparent effect of Buffer A of the Exo-spin kit on cell viability). *p<0.05, **p<0.01, ***p<0.001, ****p<0.0001, n.s.: p>0.05, two-tailed Student's t-test.

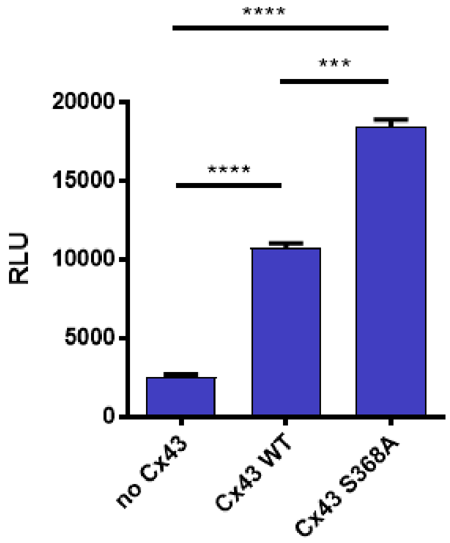

Supplementary Figure 6. Comparison of Cx43 WT and Cx43 S368A. HEK-293T cells were transfected with the following plasmid together with the other EXOtic devices (pDB60, pSA462, pSA465, pRVG-Lamp2b). Mock: EYFP-C1 (P_{hCMV}-EYFP-pA), Cx43 WT: pDB67 (P_{hCMV}-Cx43-pA), Cx43 S368A: pDB68 (P_{hCMV}-Cx43 S368A-pA). The cell culture supernatant containing engineered exosomes was applied to target HEK-293T cells expressing CHRNA7 and nluc expression was assayed at 24 hr after the medium transfer. Data shown represents mean ± SEM of three independent experiments (n = 3). ***p<0.001, ****p<0.0001, two-tailed Student's t-test.

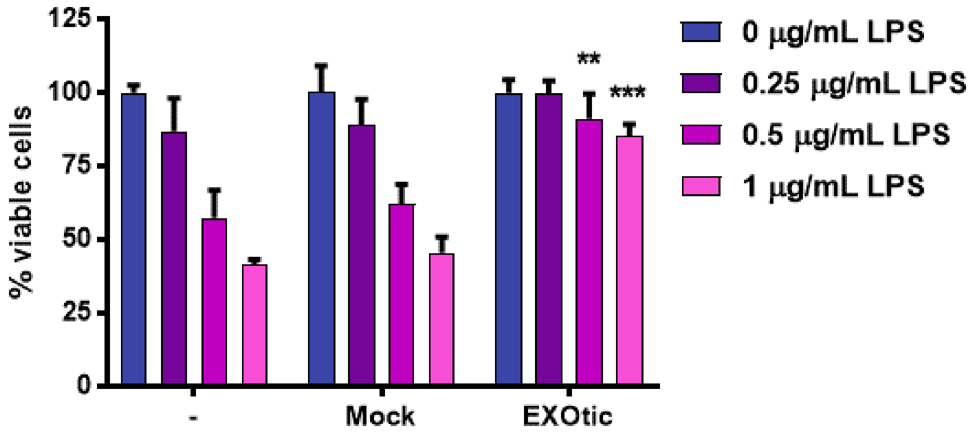

Supplementary Figure 7. Protection against LPS-induced neurotoxicity by therapeutic catalase mRNA delivery *in vitro*. Assay procedure was the same as for Fig. 3b except in the following respects: Neuro2A cells were co-cultured with microglial BV2 cells at a ratio of 5:1 and the toxin was 0-1 μg/mL of LPS. Data shown represent mean ± SEM of three independent experiments (n = 3). **p<0.01, ***p<0.001 (against both – and mock condition), two-tailed Student's t-test.

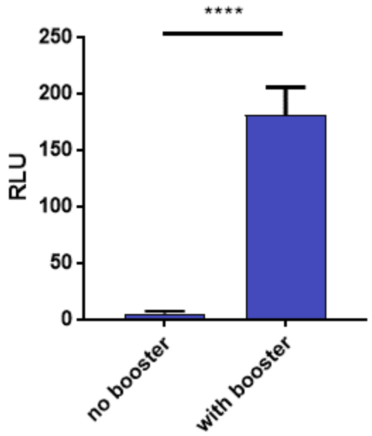

Supplementary Figure 8. CD63-nluc assay in mouse serum to check whether the exosomes secreted from implanted exosome producer cells can enter blood vessels. HEK-293T cells were transfected with pDB30 (P_{hCMV}-CD63-nluc-pA), pRVG-Lamp2b (P_{hCMV}-RVG-Lamp2b-pA), and pDB60 (P_{hCMV}-STEAP3 -IRES-SDC4-IRES-NadB fragment-pA). The engineered cells were mixed with Matrigel and implanted subcutaneously into living mice (C57BL/6J). At 24 hrs after implantation, blood was taken and serum was prepared. Then, nluc activity in the serum was assayed. Data shown represent mean ± SEM of 8 mice (n=8). ****p<0.0001, two-tailed Student's t-test.

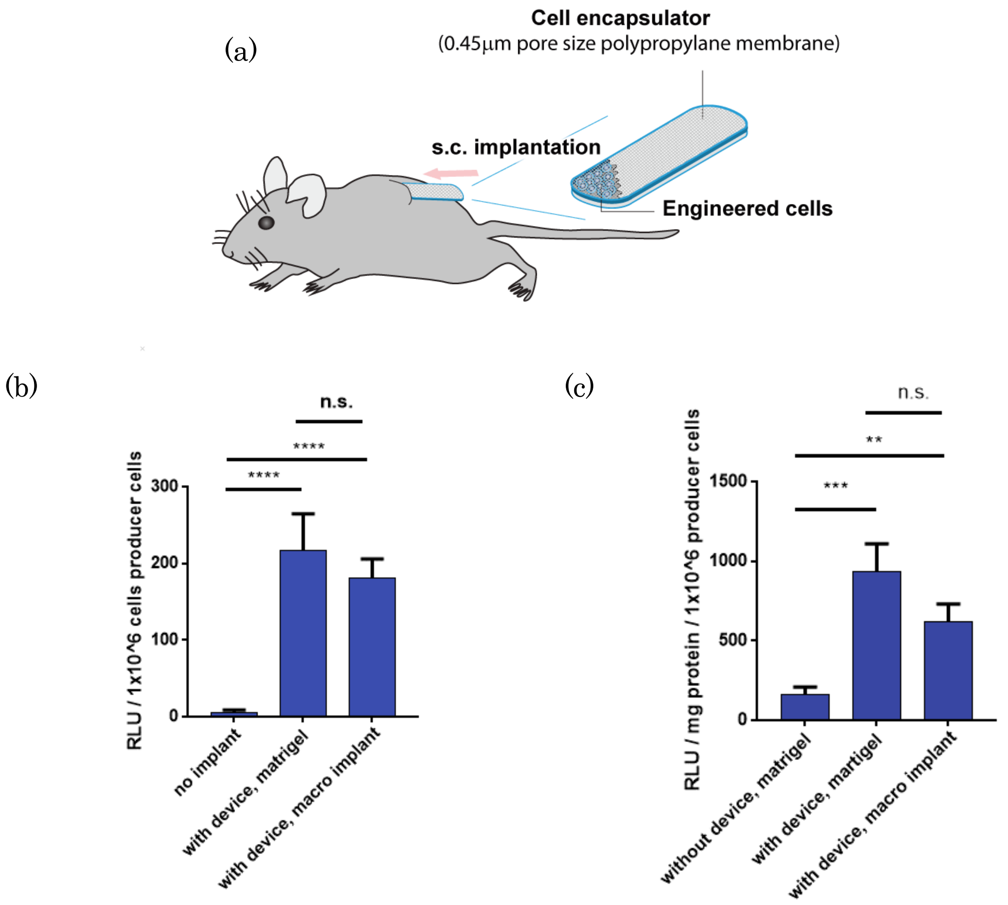

Supplementary Figure 9. In situ production of designer exosomes from exosome producer cells encapsulated in a macro encapsulator having an average pore size of 0.45 μm^2. (a) Schematic of the macro encapsulator. (b) CD63-nluc assay in mouse serum. Exosome producer cells were s.c. implanted with either Matrigel or the macro encapsulator. 24 hours later, CD63-nluc signal was monitored in the serum. (c) mRNA delivery from exosome producer cells encapsulated in a macro encapsulator. The same exosome producer cells as in Fig. 3d were prepared. 1.0×10^6 cells were encapsulated in the macro encapsulator, which was implanted subcutaneously at the back of the mice. 2 days later, brain homogenate was prepared using the same method as in Fig. 3d, and luminescence was measured. The Y axis shows the luminescence value normalized by protein concentration of the homogenate and the cell number of exosome producer cells (RLU/mg protein/1×10^6 cells of exosome producer cells). Data shown represent mean ± SEM of 8 mice (n=8). **$p<0.01$, ***$p<0.001$, ****$p<0.0001$, n.s.: $p>0.05$. two-tailed Student's t-test.

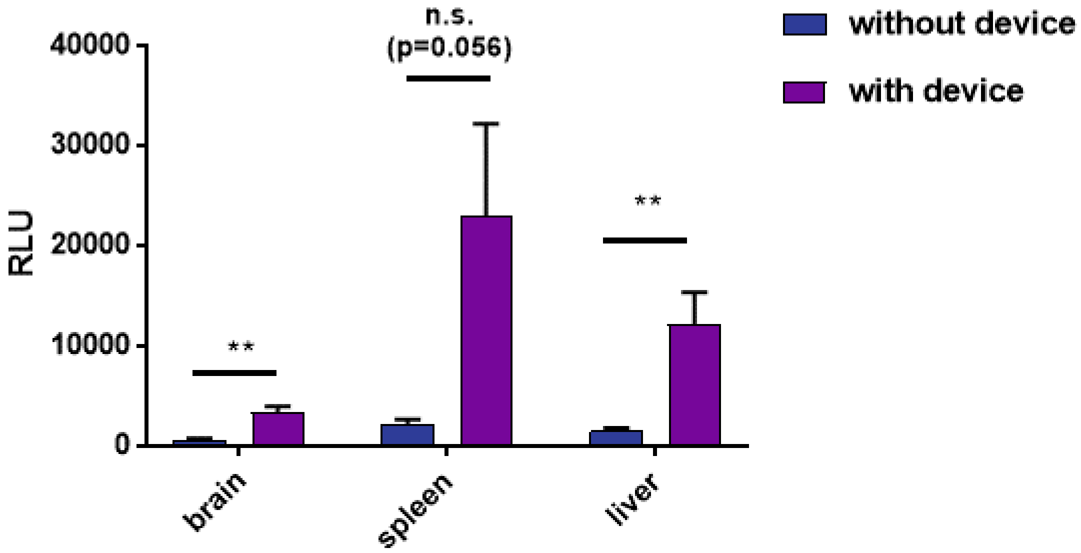

Supplementary Figure 10. Biodistribution of nluc after mRNA delivery. The mice were treated with the same procedure as in Fig. 3d. After whole-body perfusion with PBS, each organ was excised and homogenized. The homogenate was centrifuged at 9000xg for 20 min, and the nluc activity in the supernatant was assayed. At the same time, the protein concentration of the supernatants was measured with Bradford assay, and the luminescence signal (RLU) was normalized by the amount of protein. (Y axis is RLU/mg protein). Data shown represent mean ± SEM of 7-8 mice (n=7 or 8). **p<0.01, * n.s.: p>0.05. two-tailed Student's t-test.

Supplementary Table 1: Description of the plasmids used in this study.

Plasmid	Description and Cloning Strategy	Reference/Source
**pcDNA3.1(+)**	Constitutive P_{hCMV}-driven mammalian expression vector (P_{hCMV}-MCS-pA_{bGH}).	Invitrogen
**pEYFP-C1**	Constitutive EYFP expression vector (P_{hCMV}-EYFP-pA_{SV40}).	Clontech
**pTRIDENT1**	Tricistronic expression vector for multiple genes separated by IRES features. ($P_{hCMV*-1}$-MCS-IRES-MSC-IRES-pA_{SV40}).	Fussenegger et al[3]
**pRVG-Lamp2b**	Constitutive P_{hCMV}-driven RVG-Lamp2b expression vector. GNSTM glycosylation signal is added	Addgene #71924[4]
**pSA462**	Constitutive nluc expression with one C/D_{box} motif in 3'UTR (P_{hCMV}-nluc-C/D_{box}-pA_{bGH}). 1xC/D_{box} was inserted by annealing oSAE9 and oSAE10, digested with *Age*I/*Xba*I and cloned into the corresponding sites (*Age*I/*Xba*I) of pRK0.	This work
**pSA463**	Constitutive nluc expression with three C/D_{box} motifs in 3'UTR (P_{hCMV}-nluc-3xC/D_{box}-pA_{bGH}). 2xC/D_{box} was inserted by annealing oSAE11 and oSAE12, digested with *Cla*I/*Xho*I and cloned into the corresponding site (*Bst*BI/*Xho*I) of pSA462.	This work
**pSA464**	Constitutive nluc expression with five C/D_{box} motifs in 3'UTR (P_{hCMV}-nluc-5xC/D_{box}-pA_{bGH}). 2xC/D_{box} was inserted by annealing oSAE11 and oSAE12, digested with *Cla*I/*Xho*I and cloned into the corresponding site (*Bst*BI/*Xho*I) of pSA463.	This work
**pSA465**	Constitutive expression of CD63-L7Ae (P_{hCMV}-CD63-L7Ae-pA_{bGH}). L7Ae was PCR-amplified from pSA91 (Ausländer et al.[8]) using oSAE1 and oSAE2, digested with *Eco*RI/*Xba*I and cloned into the corresponding sites (*Eco*RI/*Xba*I) of pDB30.	This work
**pRK0**	Constitutive expression of nluc (P_{hCMV}-nluc-pA_{bGH}). Nanoluc was PCR-amplified from pNL1.1 (Promega) using oRK1 and oRK2, digested with *Hind*III/*Xba*I and cloned into the corresponding sites (*Hind*III/*Xba*I) of pcDNA4 V5-His B (Clontech).	This work
**pRK50**	Constitutive expression of constitutively active PI3K. (P_{hCMV}-PI3K CA-pA_{bGH}). PI3K H1047R (a gift from Weaver Alissa) was PCR-amplified using oRK68 and oRK69, digested with *Hind*III/*Xho*I and cloned into the corresponding sites (*Hind*III/*Xho*I) of pcDNA3.1(+).	This work
**pRK246**	Constitutive expression of NadB fragment (P_{hCMV}-NadB fragment-pA_{bGh}). The 3rd ORF of pDB60 was PCR amplified with oRK998 and oRK999, digested with *Hind*III/*Xba*I, and cloned into the corresponding sites (*Hind*III/*Xba*I) of pcDNA3.1(+).	This work
**pDB7**	Constitutive expression of SMPD2 (P_{hCMV}-SMPD2-pA_{SV40}).	DNASU, HsCD00438229
**pDB11**	Constitutive expression of RAB27A (P_{hCMV}-RAB27A-pA_{bGH}). RAB27A was PCR-amplified from HsCD00440409 (DNASU) using oDB4 and oDB5, digested with *Hind*III/*Xba*I and cloned into the corresponding sites (*Hind*III/*Xba*I) of pcDNA3.1(+).	This work
**pDB12**	Constitutive expression of STEAP3 (P_{hCMV}-STEAP3-pA_{bGH}). STEAP3 was PCR-amplified from HsCD00081929 (DNASU) using oDB6 and oDB7, digested with *Hind*III/*Xba*I and cloned into the corresponding sites (*Hind*III/*Xba*I) of pcDNA3.1(+).	This work
**pDB13**	Constitutive expression of SDC4 (P_{hCMV}-SDC4-pA_{bGH}). SDC4 was PCR-amplified from HsCD0074146 (DNASU) using oDB8 and oDB9, digested with *Hind*III/*Eco*RI and cloned into the corresponding sites (*Hind*III/*Eco*RI) of pcDNA3.1(+).	This work
**pDB14**	Constitutive expression of SDCBP (P_{hCMV}-SDCBP-pA_{bGH}). SDCBP was PCR-amplified from HsCD00436572 (DNASU) using oDB10 and oDB11, digested with *Bam*HI/*Xba*I and cloned into the corresponding sites (*Bam*HI/*Xba*I) of pcDNA3.1(+).	This work
**pDB17**	Constitutive expression of RAB27B (P_{hCMV}-RAB27B-pA_{bGH}). RAB27B was PCR-amplified from HsCD00434465 (DNASU) using oDB16 and oDB17, digested with *Hind*III/*Xba*I and cloned into the corresponding sites (*Hind*III/*Xba*I) of pcDNA3.1(+).	This work
**pDB18**	Constitutive expression of hnRNPA2B1 (P_{hCMV}-hnRNPA2B1-pA_{bGH}). hnRNPA2B1 was PCR-amplified from HsCD00444225 (DNASU) using oDB32 and oDB33, digested with *Eco*RI/*Xho*I and cloned into the corresponding sites (*Eco*RI/*Xho*I) of pcDNA3.1(+).	This work
**pDB30**	Constitutive expression of CD63-nluc (P_{hCMV}-CD63-nluc-pA_{bGH}). nluc was PCR-amplified from pRK0 using oDB34 and oDB35, digested with *Eco*RI/*Xba*I and cloned into the corresponding sites (*Eco*RI/*Xba*I) of pRK63.	This work

**pDB59**	Constitutive expression of STEAP3 and SDC4 connected by an IRES feature (P_{hCMV}-STEAP3-IRES-SDC4-IRES-MCS-pA_{SV40}). STEAP3 was PCR-amplified from pDB12 using oDB71 and oDB72, digested with *Eco*RI and *Hind*III and cloned into the corresponding sites (*Eco*RI/*Hind*III) of pTRIDENT1. SDC4 was PCR-amplified from pDB13 using oDB73 and oDB85, digested with *Not*I and *Cla*I and cloned into the corresponding sites (*Not*I/*Cla*I) of pTRIDENT1-STEAP3. The hCMV-promoter was cut out from pcDNA3.1(+) with *Ssp*I/*Eco*RI and cloned into the corresponding sites (*Ssp*I/*Eco*RI) of pTRIDENT1-STEAP3-SDC4.	This work
**pDB60**	Constitutive expression of STEAP3, SDC4 and NadB fragment connected by IRES features (P_{hCMV}-STEAP3-IRES-SDC4-IRES-NadB fragment-pA_{SV40}). NadB fragment was unexpectedly PCR amplified from DNASU HsCD00444272 with oDB75 and oDB127. (The fragment encodes MPLFA-E.Coli NadB (214-514)). The fragment was digested with *Xho*I/*Pac*I and was cloned into the corresponding site of pDB59.	This work
**pDB63**	Constitutive expression of CHRNA7 (P_{hCMV}-CHRNA7-pA_{bGH}).	Addgene #62276
**pDB67**	Constitutive expression of Cx43 (P_{hCMV}-Cx43-pA_{bGH}). Cx43 was PCR-amplified from HsCD00434989 using oDB93 and oDB94, digested with *Hind*III and *Xba*I and cloned into the corresponding sites (*Hind*III/*Xba*I) of pcDNA3.1(+).	This work
**pDB68**	Constitutive expression of Cx43(S368A) (P_{hCMV}-Cx43(S368A)-pA_{bGH}). Cx43(S368A) was PCR-amplified from HsCD00434989 using oDB93 and oDB95, digested with *Hind*III/*Xba*I and cloned into the corresponding sites (*Hind*III/*Xba*I) of pcDNA3.1(+).	This work
**pDB96**	Constitutive expression of CD9-nluc (P_{hCMV}-CD9-nluc-pA_{bGH}). CD9 was PCR-amplified from HsCD00000561 using oDB136 and oDB137, digested with *Hind*III/*Eco*RI and cloned into the corresponding sites (*Hind*III/*Eco*RI) of pDB30.	This work
**pDB129**	Constitutive expression of Catalase with a C/D_{box} motif in 3'UTR (P_{hCMV}-Catalase-C/D_{box}-pA_{bGH}). Catalase was PCR-amplified from HsCD00398454 with oDB199 and oDB200, digested with *Nhe*I and *Xba*I and cloned into the corresponding sites (*Nhe*I/*Xba*I) of pSA462.	This work

Supplementary Table 2: Table of oligonucleotides used for cloning.

**oDB4**	TTATACAAGCTTGCCACCATGTCTGATGGAGATTATGATTACCTC
**oDB5**	ATACTCTAGATCAACAGCCACATGCCCCTTTCTCCTTTTC
**oDB6**	ATACAAGCTTGCCACCATGCCAGAAGAGATGGACAAGCCAC
**oDB7**	ATACTCTAGATCATACGTGGCTCGTCTTCTCGG
**oDB8**	TTGTATACAAGCTTGCCACCATGGCCCCCGCCCGTCTGTTC
**oDB9**	GGCGGAATTCTCACGCGTAGAACTCATTGGTGGG
**oDB10**	ATCCGGATCCGCCACCATGTCTCTCTATCCATCTCTCGAAG
**oDB11**	ATACTCTAGATTAAACCTCAGGAATGGTGTGGTCCA
**oDB16**	TTATACAAGCTTGCCACCATGACCGATGGAGACTATGATTATCTG
**oDB17**	ATACTCTAGACTAGCAGATACATTTCTTCTCTGGTGG
**oDB32**	ATACGAATTCGCCACCATGGAGAGAGAAAAGGAACAGTTCC
**oDB33**	ATATCTCGAGTCATTGGACCGTAGTTAGAAGGTTGCT
**oDB34**	ATAGGAATTCGGCGGAGGCGGGTCCATGGTCTTCACACTCGAAGATTTCG
**oDB35**	ATACTCTAGATTACGCCAGAATGCGTTCGCAC
**oDB71**	TATGAATTCGCCACCATGCCAGAAGAGATG
**oDB72**	TATAAGCTTTCATACGTGGCTCGTCTTCTCG
**oDB73**	TATGCGGCCGCATGGCCCCCGCCCGTCTG
**oDB75**	TATCTCGAGATGCCTCTTTTTGCCACCAATCC
**oDB85**	TCCATCGATCTACGCGTAGAACTCATTGGTG
**oDB93**	TACAAGCTTGCCACCATGGGTGACTGGAGCGCCTTA
**oDB94**	TATTCTAGATCAGATCTCCAGGTCATCAGGCC
**oDB95**	TATTCTAGATCAGATCTCCAGGTCATCAGGCCGAGGTCTGCTGGCGGCACG
**oDB127**	CTCTTAATTAATCAGGAAGGGCTGGGTTTGCGGATGG
**oDB134**	TATACTAGTGGCGGAGGCGGGTCCATG
**oDB136**	TATAAGCTTGCCACCATGCCGGTCAAAGGAGGCAC
**oDB137**	TATGAATTCCAAGACCATCTCGCGGTTCC
**oDB170**	TATACTAGTGATCTCCAGGTCATCAGGCCG
**oDB196**	TATGCGGCCGCTCACTTCTGCAGGCCCTTGATCTTTTC

**oDB199**	TATGCTAGCGCCACCATGGCTGACAGCCGGGATCCCGCCAG
**oDB200**	TCTTCTAGACTACAGATTTGCCTTCTCCCTTGCCGCCAAG
**oDB260**	TATCTCGAGAGCAGTGTGGTTGTTTTTCGGGTAATTC
**oDB261**	TATTCCGGATCGATCCTGGACAGGATGCAGGC
**oDB262**	TCGAGTACACCATTTGGATGCCCGAGAATCCGAGACCAGGGACACCTTGTGACATTTTTACCAA TAGCAGAGGGAAGAGAGCATCCAACGGGT
**oDB263**	CCGGACCCGTTGGATGCTCTCTTCCCTCTGCTATTGGTAAAAATGTCACAAGGTGTCCCTGGTCT CGGATTCTCGGGCATCCAAATGGTGTAC
**oDB270**	TATCTCGAGATCTCTTTTGCAAGTGTAAACAGCACTCAAG
**oDB271**	TATTCCGGACCCTGCCCACATCAGGTGGAC
**oRK1**	CATAAAGCTTGCCACCATGGTCTTCACACTCGAAGATTTCGTTGGGG
**oRK2**	ATTGTCTAGATTACGCCAGAATGCGTTCGCACAGC
**oRK68**	TAATAAGCTTGCCACCATGCCTCCAAGACCATCATCAGGTGAACTGTGG
**oRK69**	TACGCTCGAGTCAGTTCAATGCATGCTGTTTAATTGTGTGGAAGATCCAATCC
**oRK70**	GATGAAGCTTGCCACCATGGCGGTGGAAGGAGGAATGAAATGTG
**oRK71**	TACCGAATTCCATCACCTCGTAGCCACTTCTGATACTCTTCACG
**oRK998**	CGATAAGCTTGCCACCATGCCTCTTTTTGCCACCAATCCGG
**oRK999**	ATTCTCTAGATTATCTGTTTATGTAATGATTGCCGGGGGAAAGG
**oSAE1**	ATGGAATTCGGCGGAGGCGGGTCCATGTACGTGCGCTTCGAGGTG
**oSAE2**	ACGGGCCCTCTAGATCACTTCTGCAGGCCCTTG
**oSAE9**	TCGTCTAGATGCAAAATAGACTTTAGAGGGTACCGTGATCCGAAAGGTGAGTACCCTGCAACCT CGACTTCGAACTCGAGACCGGTCGA
**oSAE10**	TCGACCGGTCTCGAGTTCGAAGTCGAGGTTGCAGGGTACTCACCTTTCGGATCACGGTACCCTCT AAAGTCTATTTTGCATCTAGACGA
**oSAE11**	CGAGCGATCGATGCCATCCGTGATCCGAAAGGTGAGATGGCTGCAGGTCGACCACTTCGGGTAC CGTGATCCGAAAGGTGAGTACCCTGCAGGTCGACTTCGAACTCGAG CGATCG
**oSAE12**	CGATCGCTCGAGTTCGAAGTCGACCTGCAGGGTACTCACCTTTCGGATCACGGTACCCGAAGTG GTCGACCTGCAGCCATCTCACCTTTCGGATCACGGATGGCATCGATCGCTCG

Supplementary references

S1. Bussi, C.; Peralta Ramos, J. M.; Arroyo, D. S.; Gaviglio, E. A.; Gallea, J. I.; Wang, J. M.; Celej, M. S.; Iribarren, P., Autophagy down regulates pro-inflammatory mediators in BV2 microglial cells and rescues both LPS and alpha-synuclein induced neuronal cell death. *Sci. Rep*. 2017, 7, 43153.

S2. Lathuiliere, A.; Cosson, S.; Lutolf, M. P.; Schneider, B. L.; Aebischer, P., A high-capacity cell macroencapsulation system supporting the long-term survival of genetically engineered allogeneic cells. *Biomaterials* 2014, 35 (2), 779-791.

S3. Fussenegger, M.; Mazur, X.; Bailey, J. E., pTRIDENT, a novel vector family for tricistronic gene expression in mammalian cells. *Biotechnol. Bioeng.* 1998, 57 (1), 1-10.

S4. Hung, M. E.; Leonard, J. N., Stabilization of exosome-targeting peptides via engineered glycosylation. *J. Biol. Chem.* 2015, 290 (13), 8166-72.
